# Guillain-Barré syndrome following the second dose of COVID AstraZeneca vaccine in a 78-year-old male: a case report from Nepal

**DOI:** 10.1097/MS9.0000000000000193

**Published:** 2023-02-17

**Authors:** Bimarsh Acharya, Sabin KC, Shailendra Karki, Pratima Thapa, Pooja KC

**Affiliations:** a KIST Medical College and Teaching Hospital, Lalitpur, Gwarko; b Gandaki Medical College, Pokhara; c Kathamandu Medical College, Sinamangal, Kathmandu; d Pokhara Academy of Health and Science, Pokhara, Nepal

**Keywords:** AstraZeneca, case report, Guillain-Barré syndrome, post-COVID-19 vaccination

## Abstract

**Introduction and Importance:**

Guillain-Barré syndrome (GBS) is a rare acute idiopathic demyelinating polyneuropathy that causes bilateral, symmetrical, and progressive weakness of muscles. AstraZeneca vaccine is a genetically modified spike glycoprotein vaccine of an adenovirus vector. GBS following the second dose of the AstraZeneca vaccine dose is rare and not frequently noted.

**Case Presentation:**

A 78-year-old male presented to the hospital with complaints of bilateral weakness of the lower limbs over 4 days following the second dose of the AstraZeneca vaccine. On examination, the power and tone of the limbs were diminished. The sensitivity pinprick test revealed low sensitivity in the right lower limb than in the left lower limb. Nerve conduction studies revealed acute inflammatory demyelinating polyneuropathy and the patient was diagnosed with GBS. After admission, the patient was successfully treated with intravenous immunoglobulins along with physiotherapy.

**Clinical Discussion:**

GBS can be diagnosed clinically with nerve conduction studies and Brighton’s criteria. The robust causal relationships between COVID-19 infections, COVID-19 vaccination, and GBS are still unclear. The evaluation of the potential association and risk of GBS with vaccines warrants the need for precise post-vaccination surveillance measures and results.

**Conclusion:**

Only a few cases of GBS following the second dose of AstraZeneca are reported so far and there is a need for strong and accurate diagnosis of the disease and proper post-vaccination surveillance for the evaluation of risk associated with COVID vaccines.

HIGHLIGHTS

- Guillain-Barré syndrome (GBS) is a rare disease that causes bilateral, symmetrical progressive weakness of the muscles.
- Reports of GBS following AstraZeneca COVID vaccination warrants the need for post-vaccination surveillance globally.
- Nerve conduction studies and Brighton’s criteria can be used to diagnose GBS.
- GBS can be successfully managed with Intravenous immunoglobulin therapy.

## Introduction

AstraZeneca is a recombinant, nonreplicative Spike (S) glycoprotein vaccine created using chimpanzee adenovirus vector, in a genetically modified human embryonic kidney (HEK 293) cell lines, with reported efficacy of 70.4%. after two standard doses^1^. Cerebral venous sinus thrombosis; thrombocytopenia, GBS, and acute transverse myelitis have been reported following the AstraZeneca vaccine^2^. About 18.6 million people took the first dose and 7.24 million people have completed two standard doses in Nepal by the September of 2022. To our knowledge, there is the first case of GBS following the second dose of the COVID-19 AstraZeneca vaccine from Nepal. This case report has been reported in line with the SCARE Criteria^3^.

## Case presentation

Our 78-year-old male presented with the complaint of bilateral lower limb swelling, weakness, and tingling sensation of finger and toes for 4 days, following 15 days after the second dose of AstraZeneca vaccination. Weakness was acute in onset and gradually progressed to the upper limbs within a week and was associated with difficulty in sitting, standing, and walking activities. He had no history of trauma, headache, photophobia, double vision, abnormal body movements, loss of consciousness, bowel or bladder incontinence, and respiratory or gastrointestinal illness. However, there is a past history of Hashimoto thyroiditis, hypertension, diabetes mellitus, and chronic obstructive pulmonary disease, and have been taking medicines for these conditions.

On examination vitals were stable (blood pressure 160/80 mm, respiratory rate 18/min, pulse rate 86 bpm, and SpO_2_ 96%). The Glasgow Coma Scale was recorded at 15/15 and all cranial nerves were intact. Muscle bulk was normal but the tone and power were diminished. Power of both the shoulder and elbow 3/5, left: wrist flexor 2/5, extensor 3/5, handgrip strength 50% and right: wrist flexor 2/5, extensor 2/5, handgrip strength 60%, left: hip flexor, abductor, and extensor 2/5, knee flexor and extensor 3/5, dorsal flexion, plantar flexion, and great toe 1/5, right: hip flexor abductor and extensor 2/5, knee flexor 2/5, extensor 3/5, dorsiflexion and plantar flexion 1/5 and great toe 1/5. Sitting balance test: static was good and dynamic was fair and reflexes were preserved. The sensation was decreased on the right lower limb more than on the left lower limb to the pinprick test.

Laboratory investigation showed hemoglobin (12.7 g/dl), packed cell volume (38.7%), increased C-reactive protein (66.6 mg/l), hypoalbuminemia (2.90 g/dl), aspartate aminotransferase (103 U/l), and elevated alkaline phosphatase (488 U/l). The patient was hyperkalemic (5.40 mmol/l) and his serum urea was 47 mg/dl. Also, the random blood sugar was 175 mg/dl and free T3 was 1.70 pg/ml. Cerebrospinal fluid showed adenosine deaminase 2.86 Ul, glucose 60 mg/dl, and total protein of 53 mg/dl. No significant findings on the computed tomography scan. Nerve conduction findings were consistent with acute inflammatory demyelinating polyneuropathy.

**Figure 1:**
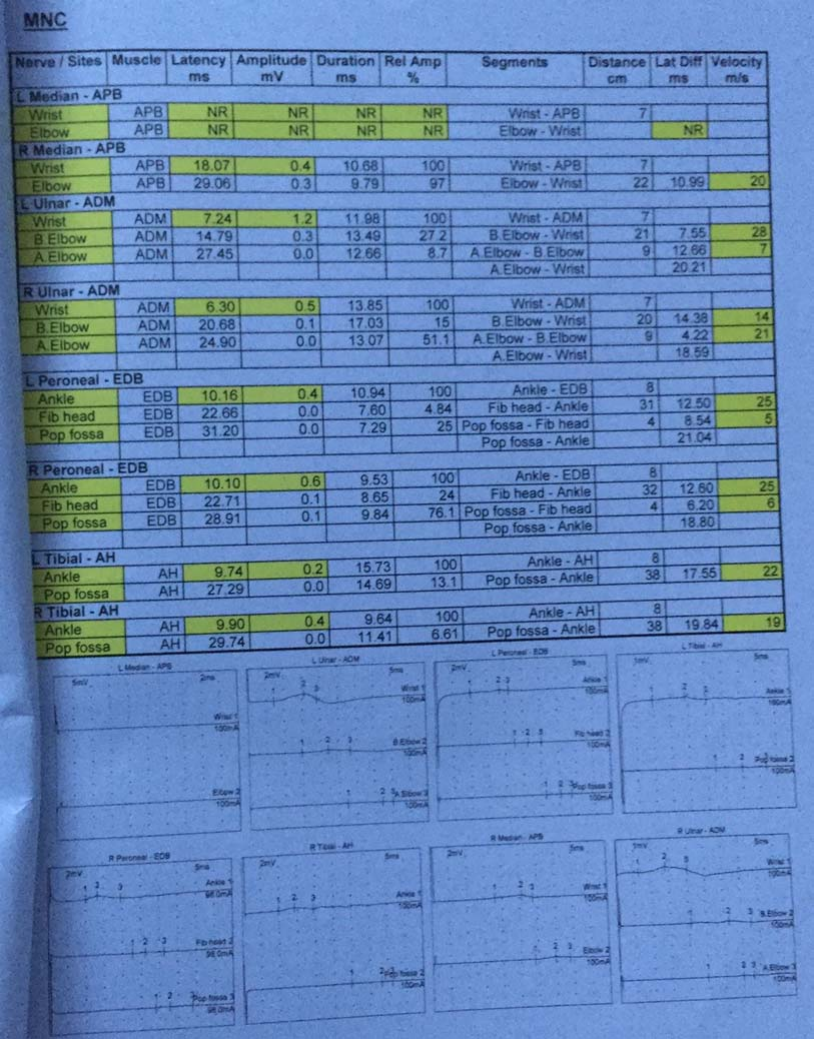
Motor nerve conduction (MNC).

**Figure 2:**
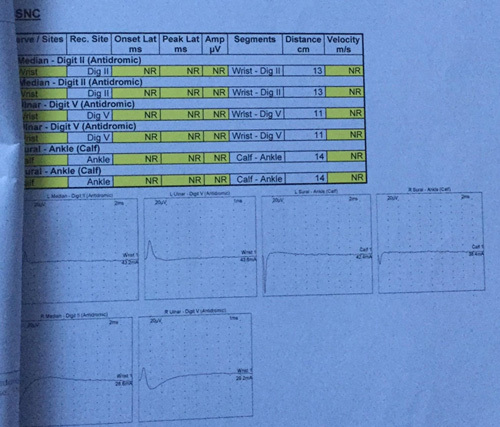
Sensory nerve conduction (SNC).

Nerve conduction study tables show the electrophysiological evidence of sensorimotor axonomylenic polyneuropathy of a severe degree along with the abnormal motor, sensory, and F-wave patterns in Figures 1 and 2, respectively.

**Figure 3:**
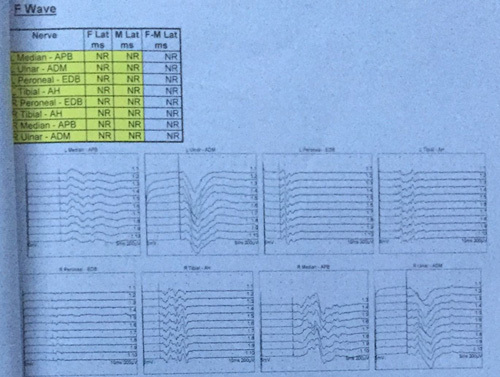
F wave.

With the help of clinical findings and nerve conduction findings, Brighton’s criteria patient was diagnosed with GBS and was admitted to the Mediciti Hospital Kathmandu. Five doses of Intravenous immunoglobulin were commenced from the day of admission and his condition gradually improved afterward. Along with the medical treatment patient also underwent physiotherapy. He was discharged after 10 days following the treatment with immunoglobulins. With three follow-ups in between and 6 months later, he finally recovered with no neuromotor or sensory dysfunction at the time. Our patient believes that his conditions were due to COVID vaccination and also satisfied with the treatment provided in Figure 3.

## Discussion

Along with other parts of the world, Nepal has also been one of the prime victims of the COVID pandemic. A total of 12 016 deaths were recorded from 3 January 2020 to September 2022, and more than 9000 cases have been recorded. Overall, 22 324 933 (76.61%) of the population have been fully vaccinated throughout this time^4^.

Our patient developed the symptoms of bilateral limb weakness 15 days after following the second dose with AstraZeneca (ChAdOx1 nCoV-19). There have been reports of GBS following AstraZeneca and other post-COVID vaccinations^5^,^6^. Reporting of such cases should be scrutinized with accurate diagnosis and strong post-vaccination surveillance systems for the proper evaluation of risk.

The known etiopathogenesis of GBS is molecular mimicry of pathogen-borne antigens, which results in the formation of antiganglionic cross-reactive antibodies and activation of a complement system that targets the gangliosides. The type of infection and the specific antiganglioside antibody defines the subtype of GBS. Our electrophysiological findings were consistent with acute inflammatory demyelinating polyneuropathy which results due to the invasion of myelin by macrophages due to activation of the complement system and membrane attack complex formation on the outer surface of Schwann cells^7^. Some evidence suggests that individuals with T-cell glycolipid CD1 polymorphisms are more susceptible to GBS^8^.

GBS by vaccine virus or vaccine-associated products occurs possibly due to the insertion of virus-specific polypeptides from neighboring cells into host cell membranes damaged by the virus resulting in the humoral or cell-mediated mechanism against myelin antigen in circulations There is only a little evidence of vaccine-associated GBS with Swine flu vaccine 1976, older formulation Rabies vaccines cultured in mammalian brain tissues, Quadrivalent conjugate meningococcal vaccine (MCV4)^9^.

Vaccine-associated GBS is considered a relevant diagnosis if received up to 4 weeks before the onset of symptoms^10^. Also, the Brighton criteria is significant to diagnose GBS in clinical settings^11^. Our patient was observed at the level of 4 as per the criteria. According to a nerve conduction study (*n*=93), 70% of Hadden’s and 38% of Rajabally’s criteria had primary demyelinating characteristics for GBS^12^. Our case also follows the same results as shown in Figure 1. In two different case series regarding GBS following post-COVID vaccination, all patients were in their fifth to seventh decade of life and the common presentations occurring within 3 weeks of vaccination were bifacial weakness with paresthesia’s variants^5^,^6^,^13^. It took 6 months for our patient to return to his normal activities. A systematic review of GBS in association with COVID-19 vaccination reveals 85% partial recovery among the affected individuals^14^.

Although 900–2200 per billion people are expected to develop GBS within 6 weeks of receiving the first dose of vaccine and 1500–3700 per billion within a 10-week period from following the second dose of vaccination, no significant relation between COVID vaccines and GBS is established from available data^15^.

## Conclusion

Vaccines have proven to be our most important tool to fight the COVID pandemic and very few cases of GBS following the second dose of AstraZeneca and other vaccines are reported so far. Our case was presented with acute onset of bilateral limb weakness which was treated with the help of intravenous immunoglobulin and physiotherapy. More studies and evidence are required to signify the causality among the reported cases which would eventually assist to prevent the further burden of the COVID pandemic.

## Provenance and peer review

Not commissioned, externally peer-reviewed.

## Ethical approval

Since this is a case report. Ethical approval is not required.

## Consent

Written informed consent was obtained from the patient for the publication of this case report and accompanying images. A copy of the written consent is available for review by the Editor-in-Chief of this journal on request.

## Sources of funding

None.

## Authors’ contribution

B.A. wrote the manuscript and edited the manuscript. S.K.C. worked on data collection and also took written consent for the publication of the case report from the patient. S.K., P.T., and P.K.C. were involved in the review process of the literature and preparation of the manuscript. All the authors individually did the final proofreading of the manuscript before submission.

## Conflicts of interest disclosure

All authors declare that they have no any conflicts of interest.

## Research registration unique identifying number (UIN)

None.

## Guarantor

Bimarsh Acharya.
